# Genome-wide association study for frozen-thawed sperm motility in stallions across various horse breeds

**DOI:** 10.5713/ab.21.0504

**Published:** 2022-03-03

**Authors:** Elena V. Nikitkina, Natalia V. Dementieva, Yuri S. Shcherbakov, Mikhail M. Atroshchenko, Andrei A. Kudinov, Oleg I. Samoylov, Marina V. Pozovnikova, Artem P. Dysin, Anna A. Krutikova, Artem A. Musidray, Olga V. Mitrofanova, Kirill V. Plemyashov, Darren K. Griffin, Michael N. Romanov

**Affiliations:** 1 Russian Research Institute for Farm Animal Genetics and Breeding – Branch of the L. K. Ernst Federal Science Center for Animal Husbandry, Tyarlevo, Pushkin, St. Petersburg, 196625, Russia; 2 All-Russian Research Institute for Horse Breeding, Rybnovsky District, Ryazan Oblast, 391105, Russia; 3 School of Biosciences, University of Kent, Canterbury CT2 7NJ, UK

**Keywords:** Candidate Genes, Cryopreservation, *Equus caballus*, Single Nucleotide Polymorphisms (SNPs), Sperm, Stallion

## Abstract

**Objective:**

The semen quality of stallions including sperm motility is an important target of selection as it has a high level of individual variability. However, effects of the molecular architecture of the genome on the mechanisms of sperm formation and their preservation after thawing have been poorly investigated. Here, we conducted a genome-wide association study (GWAS) for the sperm motility of cryopreserved semen in stallions of various breeds.

**Methods:**

Semen samples were collected from the stallions of 23 horse breeds. The following semen characteristics were examined: progressive motility (PM), progressive motility after freezing (FPM), and the difference between PM and FPM. The respective DNA samples from these stallions were genotyped using Axiom Equine Genotyping Array.

**Results:**

We performed a GWAS search for single nucleotide polymorphism (SNP) markers and potential genes related to motility properties of frozen-thawed semen in the stallions of various breeds. As a result of the GWAS analysis, two SNP markers, rs1141327473 and rs1149048772, were identified that were associated with preservation of the frozen-thawed stallion sperm motility, the relevant putative candidate genes being NME/NM23 family member 8 (*NME8*), olfactory receptor family 2 subfamily AP member 1 (*OR2AP1*), and olfactory receptor family 6 subfamily C member 4 (*OR6C4*). Potential implications of effects of these genes on sperm motility are herein discussed.

**Conclusion:**

The GWAS results enabled us to localize novel SNPs and candidate genes for sperm motility in stallions. Implications of the study for horse breeding and genetics are a better understanding of genomic regions and candidate genes underlying stallion sperm quality, and improvement in horse reproduction and breeding techniques. The identified markers and genes for sperm cryotolerance and the respective genomic regions are promising candidates for further studying the biological processes in the formation and function of the stallion reproductive system.

## INTRODUCTION

There is a growing interest in the preservation of genetic material from stallions with outstanding phenotypic traits using cryopreservation of spermatozoa [1–3]. Over the past decades, sperm cryopreservation is one of the most widely used methods to preserve biological material in domestic animals (e.g., [4–6]) that is also used as one of gene pool conservation strategies (e.g., [7–9]). However, stallion semen is less resistant to ultra-low temperatures as compared, for example, to bull semen. Importantly, as one of the key targets of selection, sperm quality and cryotolerance in stallions have a high individual variation that depends on both environmental and genetic factors [2, 3,10]. To date, horse breeding involves a widespread use of artificial insemination (with the exception of thoroughbred racehorses), and that is why high quality of cryopreserved semen is vital and pivotal [3,11,12].

Genome organization in sperm is functionally instrumental for controlling fertilization and early developmental processes in animals [13–15]. Determination of genetic factors affecting sperm quality indicators and sperm cryotolerance is therefore of great significance, and certain candidate genes have been found to be associated with male fertility traits and sperm quality after thawing. These genes include, for example, testis-sperm specific FKBP prolyl isomerase family member 6 (inactive) (*FKBP6*), a candidate for impaired acrosome reaction [16,17], phospholipase C zeta 1 (*PLCZ1*) [18], cysteine-rich secretory protein 3 (*CRISP3*) [19,20], and some others genomic variants [10, 21,22]. Spermatozoa progressive motility (PM) measured as speed of forward progression with flagellar movement (see for review [23,24]) is one of the most important semen quality properties before freezing and after thawing. However, the relationship between the molecular architecture of the genome, on the one hand, and mechanisms of sperm formation and their preservation after thawing, on the other, is poorly understood and requires further detailed investigation [25].

In this regard, the aim of the present investigation was to perform a genome-wide association study (GWAS) for genomic variants relevant to sperm motility of cryopreserved semen in stallions across various horse breeds using a high density single nucleotide polymorphism (SNP) chip. As a result, we were able to identify a few suggestive SNP markers and relevant candidate genes that are worthy of further research and applications in horse breeding.

## MATERIALS AND METHODS

### Animals and sample collection

Sampling procedure was approved by the Russian Research Institute of Farm Animal Genetics and Breeding (RRIFAGB) – Branch of the L. K. Ernst Federal Science Centre for Animal Husbandry (Protocol No. 2020/2), adhered to and performed in accordance with the appropriate ethical guidelines (Law of the Russia Federation on Veterinary Medicine No. 4979-1 dated 14 May 1993). The authors declare that stallion semen samples were properly collected by trained personnel following strict veterinary requirements and keeping animal discomfort and stress to a minimum.

**Figure 1:**
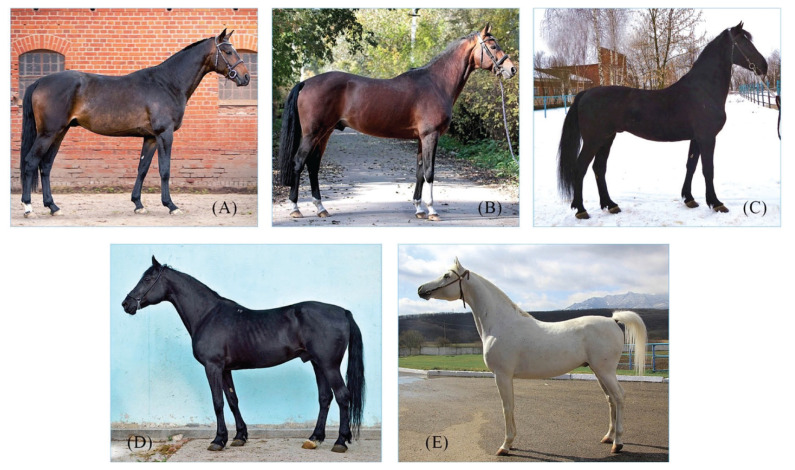
Examples of individual stallions used in the present genome-wide association study for frozen-thawed sperm motility: (A) Khitmos, of the Trakehner breed, dark bay color; (B) Santrek, of the Trakehner breed, bay color; (C) Logotip, of the Orlov Trotter, black color; (D) Vympel, of the Orlov Trotter, black color; and (E) Zhasmin, of the Arabian breed, white color.

To conduct the present GWAS, we used sperm samples from stallions kept at the All-Russian Research Institute for Horse Breeding (ARRIHB, Ryazan Oblast), the Tersk Stud Farm No. 169 (Stavropol Krai), and the Perevozsky and Pochinkovsky studs (Nizhny Novgorod Oblast). Ninety-six animals (see Figure 1 for photographs of individual stallion examples) were sampled that represented the following 23 horse breeds: Akhal-Teke, Appaloosa, Arabian, Bashkir, Budyonny (or Budennovskaya), Don, French Trotter, German Warmblood, Hanoverian, Heavy Draft crossbreds, Holsteiner, Karabakh, Orlov Trotter, Rhenish German Coldblood (or Rhenish), Russian Heavy Draft, Russian Riding, Selle Français, Soviet Heavy Draft, Standardbred, Tersk, Thoroughbred, Trakehner (or Trakehnen), and Welsh Pony. There was an average of 4.2 males per breed, with the range of respective numbers for a single breed being 1 to 35 (Table 1). All stallions were healthy and varied in terms of sperm quality after thawing; therein, stallions with poorer quality were also included in the study.

Semen was collected at least three times from each stallion using an artificial vagina. Collection of semen samples, freezing and thawing were carried out by one same group of researchers in the spring-summer period. A total of 288 semen samples, or three ejaculates from a stallion, were analyzed. There was an average of 12.5 samples per breed, the respective numbers per breed being ranged between 3 and 105. Where suitable for certain analyses, we combined samples from breeds with close breed characteristics into larger breed groups or removed few very small sized breeds to test if this could increase significance and accuracy of the obtained sperm parameters and comparisons.

### Semen examination

Sperm was diluted at 1:3 (v/v) ratio with lactose-chelate-citrate-yolk (LCCY) medium containing 3.5% glycerin and frozen according to a standard technology (standard operating procedure) used at the ARRIHB and described in detail elsewhere [11,26–28]. Briefly, four-cornered aluminum tubes were used to package the diluted semen. After filling in a tube with 18 mL of diluted semen, the dimensions of the tube were as follows: length, 105 mm; width, 35 mm; and thickness, 4.5 mm. The frozen sperm concentration was 45 to 50 million/mL.

**Figure 2:**
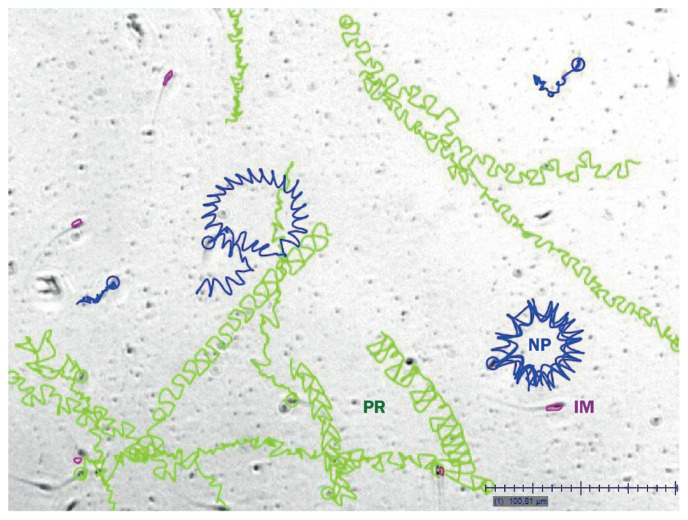
Example of the computer-assisted semen analysis (CASA) using a stallion semen sample. Sperm tracks generated by CASA: PR, progressive (green) tracks of individual sperms; NP, non-progressive (blue) tracks; and IM, immotile (or dead) sperm (red dots).

Fresh sperm PM and post-thaw forward progressive motility (FPM) were measured in percentage of actively moving spermatozoa using a computer-assisted semen analysis (CASA [24]; Figure 2). The appropriate CASA system (ArgusSoft Ltd., St. Petersburg, Russia) and a Motic BA410 microscope (Motic, Hong Kong, China) were employed for this purpose. Comparison of mean values of motility traits was performed using the Student’s t-test at a significance level of p<0.05. Then, difference (DPM) between PM and FPM values was calculated as suggested elsewhere [29] and used for the subsequent GWAS analysis.

To estimate descriptive statistics, motility data processing was performed using Microsoft Excel. Differences in PM, FPM, and DPM values between individual breeds and breed groups were evaluated for significance using the R software v. 4.1.0 [30], and the respective boxplots were produced using ggplot2 package [31].

Principal component analysis (PCA) plots and correlations were inferred from the sperm PM data using R and libraries for the R environment [30]. Based on sperm motility data before and after freezing, distribution of the studied 23 horse breeds was also tested with the web tools Phantasus [32] and ClustVis [33].

### Single nucleotide polymorphism genotyping

DNA was isolated from frozen semen samples using the phenol/chloroform method. DNA samples were genotyped using an Affymetrix high density chip, Axiom Equine Genotyping Array (Thermo Fisher Scientific, Waltham, MA, USA). DNA samples with genotyping quality more than 98% at SNP loci were selected for further examination. The SNP selection was carried out using the PLINK 1.9 software program [34] and minor allele frequency (MAF) >0.05. After quality control, 306,522 variants were available for the further analysis.

### Genome-wide association study analysis

The GWAS were performed using EMMAX software [35] and an identity-by-state kinship matrix generated by EMMAX. Phenotype of each individual for the GWAS was determined by averaging the respective trait values from three samples per individual. To calculate effect of SNP on a trait, the following model was implemented:


Y=Xb+u+Br+e,

where *Y* is a vector of phenotypes; *b* is a SNP effect; *X* is a design matrix of SNP genotypes; *u* is a vector of additive genetic effects assumed to be normally distributed with the mean equal to 0 and (co)variance *σ*^2^*aG*, with *σ*^2^*a* being additive genetic variance and *G* being genomic relationship matrix; *Br* is a breed effect; and *e* is a vector of random residual effects.

Significance and suggestive levels for a SNP effect were set as 1.631204×10^−7^ (0.05/306,522) and 3.262409×10^−6^ (1.00/306,522), respectively. The genome-wide significance was assessed using the simpleM method in R, and calculation of effective number of independent tests was performed using M_eff_ [36].

Based on the GWAS results and using the qqman package within the R software [37], a Manhattan and quantile-quantile (Q-Q) plot graphs were produced. Genes that coinciding with a candidate SNP genomic region or being close to it were determined using the *Equus caballus* (ECA; horse) genome assembly EquCab3.0 [38]. SNP information for relevant genes was retrieved using NCBI and Ensembl genomic browsers.

## RESULTS AND DISCUSSION

### Sperm motility differences

In this study, we additionally included in the analysis stallions with poor sperm cryotolerance, but since they were from different breeds, we expected to identify loci that included markers that did not depend on breed affiliation. For this, an additional adjustment was made in the analysis of GWAS for the breed effect.

As a result of assessing the quality of semen, the 288 stallion samples were analyzed in triplicate. The same ejaculates were investigated before and after freezing. Using CASA (Figure 2), we examined sperm PM by individual stallion samples, breeds, and their groups. The results obtained by breed are shown in Table 1.

**Figure 3:**
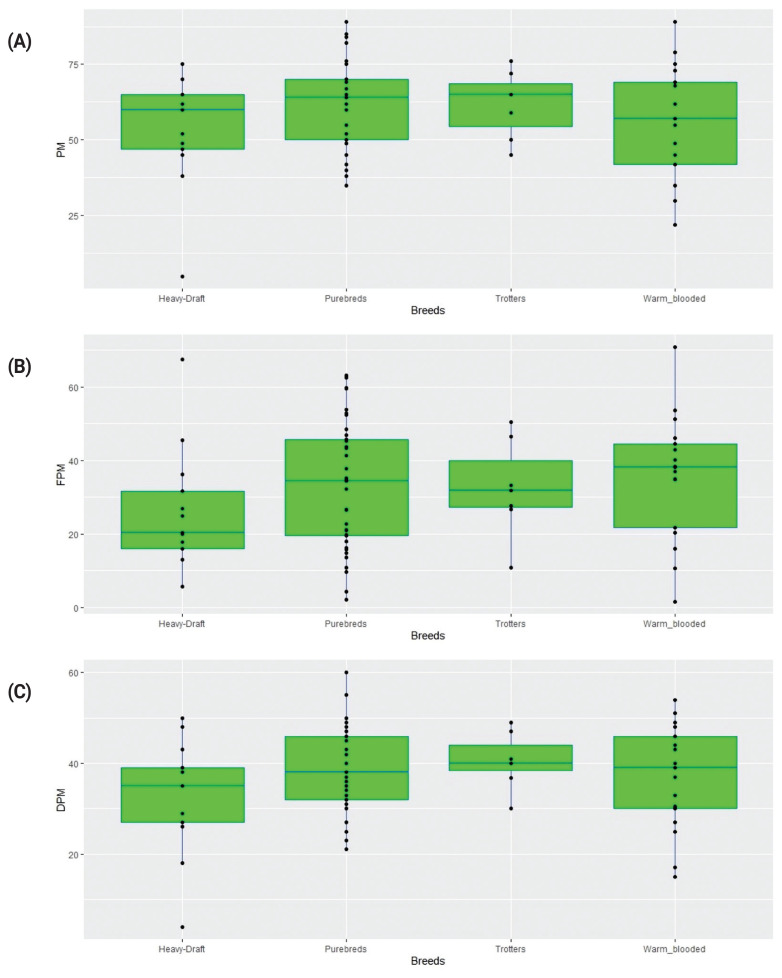
Quality parameters in stallion semen samples before and after freezing: (A) PM, fresh sperm progressive motility; (B) FPM, post-thaw forward progressive motility; and (C) DPM, difference between PM and FPM values. Breeds grouped: Heavy draft – Russian Heavy Draft, Soviet Heavy Draft; Purebreds – Akhal-Teke, Arabian, Bashkir, Budyonny, Hanoverian, Karabakh, Rhenish German Coldblood, Tersk, and Thoroughbred; Trotters – French Trotter, and Standardbred; and Warmblooded – Holsteiner, Orlov Trotter, Russian Riding, Trakehner, and Welsh Pony.

There were no significant differences in the produced values of sperm motility parameters in the stallion semen samples before and after freezing between either individual breeds (Table 1) or their groups (as represented with the respective boxplots shown in Figure 3).

**Figure 4:**
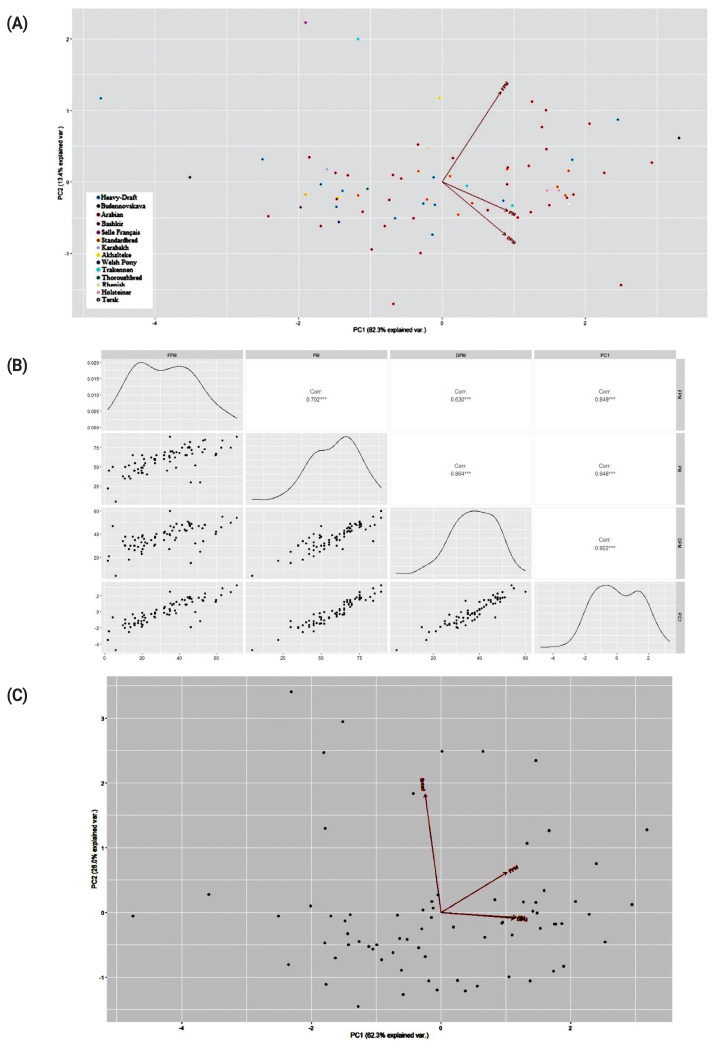
Principal component analysis (PCA) plots based on the sperm progressive motility data (A and C) and correlations between PC1 and tested factors (B). For (A) and (B), three factors were used: progressive motility (PM), post-thaw forward progressive motility (FPM), and difference (DPM) between PM and FPM values. For (C), the breed factor was also tested as the fourth eigenvector. Breeds analyzed: Akhal-Teke, Arabian, Bashkir, Budyonny, Holsteiner, Karabakh, Rhenish, Selle Français, Standardbred, Tersk, Thoroughbred, Trakehner, Welsh Pony; and breed group: Heavy draft (Russian Heavy Draft + Soviet Heavy Draft).

The PCA and clustering plots using the sperm PM data did not reveal meaningful sample/breed clustering patterns (Figures 4A,C; Supplementary Figure S1A,B), although there were high correlations between PC1 and three tested sperm motility factors, i.e., PM, FPM, and DPM (Figure 4B).

Although some breeds tended to have lower cryotolerance (as estimated by DPM) in contrast to other breeds, on average, there was no significant difference in semen characteristics between the studied horse breeds/groups that might be due to a reduced sampling size of the breeds used in the study. On the other hand, there may be best sires (refiners) and worst sires within each breed, i.e., there was individual variability in most cases (as also observed in other studies [2,3,10,39]) that exceeded the differences between breeds.

Overall, breeds in horses, as in other animal species, are however unlikely to differ significantly among themselves in semen quality. These traits are too important for the overall “vitality” of a breed to be expected to deteriorate. At the same time, within a breed, the variability of these characters may well be observed. Therefore, we suggest that in general, frozen-thawed stallion sperm motility traits are maintained at the same, averaged level across the breeds, while within-breed variation can be quite pronounced as can be seen in the Figure 3 boxplots.

### Genome-wide association study implications and candidate genes

**Figure 5:**
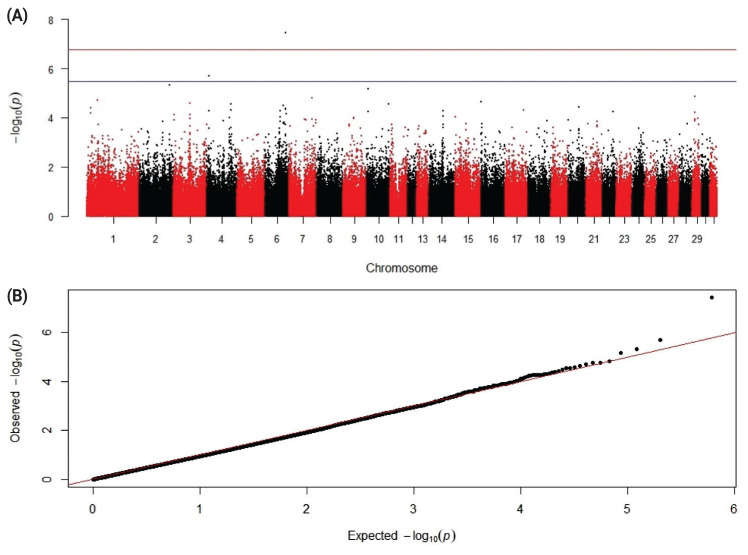
Manhattan (A) and quantile-quantile (Q-Q; B) plots resulted from the genome-wide association study for sperm DPM. Blue line corresponds to the threshold of chromosome-wide suggestive levels for a SNP effect (p<3.262409e-6), and red line represents the threshold of genome-wide significance (p<1.631204e-7). The Q-Q plot (B) shows the observed p-value plotted against the expected one. PM, progressive motility; FPM, post-thaw forward progressive motility; DPM, difference between PM and FPM values; SNP, single nucleotide polymorphism.

A search for genomic associations with DPM resulted in one significant SNP, rs1141327473 (p-value = 1.96e-06), located in the intron of the NME/NM23 family member 8 (*NME8*) gene on chromosome ECA4 (Table 2; Figure 5). The protein encoded by this gene, also known as sperm-specific thioredoxin 2 (*SPTRX2*), is probably required at the final stages of maturation of the sperm tail in the testis and epididymis, where extensive disulfide binding of fibrous proteins occurs. Mutations in this gene are involved in primary ciliary dyskinesia-6 in humans. The protein expression was found at all stages of sperm maturation in the tail [40]. Altogether, this information suggests that the *NME8* gene is involved in sperm tail formation and its function is crucial for maintaining sperm motility.

The second suggestive SNP was rs1149048772 (p-value = 3.60e-08, with frequency of the C allele being 0.12) located on ECA6 (Table 2; Figure 5). One of the candidate genes for DPM found near this SNP was olfactory receptor family 2 subfamily AP member 1 (*OR2AP1*), a member of the olfactory receptor family related to G-protein-coupled receptors (GPCR). Another gene, olfactory receptor family 6 subfamily C member 4 (*OR6C4*), is responsible for recognition and mediated by the G protein. Expression of various GPCRs on the plasma membrane of human spermatozoa suggested their involvement in the regulation of sperm motility, capacitation, and acrosome reaction [41]. Mutations in the *OR2AP1* gene were also suggested to cause adenocarcinoma of the prostate [42].

In addition, we investigated genomic associations for PM and FPM. While no significant SNP association was revealed for PM, we discovered the maximum effects for two SNPs within a region on ECA2, though insignificant ones. These were rs68590468 (p = 3.99e-06) and rs396809330 (p = 3.50e-05) in the introns of the phosphatase and actin regulator 4 (*PHACTR4*) gene, the MAF values being 0.40 (G allele) and 0.36 (C allele), respectively. We would speculate that *PHACTR4* might be a putative candidate gene for FPM, although this would require further investigation and confirmation. The *PHACTR4* gene encodes a protein that is a member of the phosphatase and actin regulator family. It is known that members of the PHACTR family inhibit the activity of protein phosphatase 1 (PP1) and interact with actin and PP1 [43]; many transcript variants of the *PHACTR4* gene have been found that encode different isoforms. PP1 plays an important role in the control of glycogen metabolism that is critical for maintaining sperm motility [44].

### Single nucleotide polymorphism genotypes associated with sperm cryotolerance

Eventually, the analysis of the semen cryotolerance parameters was carried out with regard to their association with the identified SNP genotypes in stallions (Table 3). As a result, significant differences in the DPM values were revealed. Animals with the CT and CC genotypes at the rs1141327473 locus demonstrated a better cryotolerance despite poorer PM values. Examination of the SNP rs1149048772 genotypes also showed that the best resistance to freezing was in individuals with the CC and CT genotypes.

We also determined the occurrence frequency of minor alleles at significant SNPs as shown in Table 4. As a result of comparing the data from Tables 1 and 4, it was found that at SNP rs114132747 the occurrence of the C allele in the breeds studied was associated with a better cryotolerance (DPM).

Our study suggests that cryotolerance of stallion sperm is not a breed-dependent trait, and its association with polymorphic variants in the markers (genes) affecting sperm resistance to freezing should be considered intraspecific. The prevalence of cryotolerance genotypes can be slightly shifted towards an increase in the occurrence frequency only in those breeds in which artificial insemination with cryopreserved sperm is practiced in breeding. However, this practice is not common, and for some breeds it is absolutely impossible. These breeds include all purebred breeds (e.g., Arabian, Akhal-Teke, and Thoroughbred), in which the use of artificial insemination is prohibited. In breeding farms and large breeding companies, natural mating is preferred. Thus, the accumulation of polymorphisms associated with sperm cryotolerance in horse populations is minimized due to the intensive use of artificial insemination during reproduction. Therefore, the distribution of genotypes associated with cryotolerance of stallion sperm is not under selection pressure and seems natural.

Because of these observations, we believe that it seems reasonable to consider the association of the studied SNPs with indicators of sperm resistance to cryopreservation for the sample of breeds as a whole, and not for a single breed. In particular, when analyzing the whole sample of breeds (Table 4), a significant difference in DPM (p<0.01) was identified between the CC, CT, and TT genotypes for rs1149048772. The difference in motility (DPM) between native and cryopreserved sperm associated with this SNP was lower in the CC genotyped animals (13.33%±4.98%; the motility index decreased by half) than that in the TT genotyped stallions (40.11%±1.08%; the motility declined three times from the initial one). In stallions heterozygous for the rs1149048772 substitution, i.e., with the CT genotype, the decrease in motility was 30.38%±2.17%, which is less than half the PM. A significantly higher percentage of reduced sperm motility in the TT and CT stallions after cryopreservation indicates an effect of substitution of nucleotide C for T at the studied locus on DPM.

The most favorable genotype for sperm cryotolerance at SNP rs1149048772 seemed to be the homozygous CC genotype. However, stallions with the CC genotype had a significantly reduced native sperm motility (p<0.01). Specifically, PM of the CC stallions was significantly lower and amounted to 26.66%±11.66% vs 51.75%±5.16% in the CT males and 61.92%±1.76% in the TT animals, that is, more than twice between the two homozygous genotypes. This effect can be considered as a compensating effect between sperm quality and its survival (since exposure to ultra-low temperatures can be a stress factor for reproductive cells), requiring further investigation.

A similar effect was observed for SNP rs1141327473 C>T in the *NME8* intron. Particularly, PM in the stallions with the CC genotype was only 43.71%±4.21% vs the CT and TT stallions, in which motility was 60.08%±2.64% and 65.42% ±2.39%, respectively (p<0.01). The reduction in FPM, on the contrary, was the lowest in the stallions with the CC genotype (27.00%±2.42%) and higher in the CT stallions (37.61% ±1.56%) and TT animals (42.95%±1.44%) at p<0.01.

Thus, CC genotypes at the two SNPs studied could be linked to individuals with a higher sperm cryotolerance, but negatively affected the main quality indicator, i.e., PM of native sperm.

The SNPs detected by GWAS analyses are markers that do not always play a direct functional and/or regulatory role. They may be part of haplotypes, polymorphic variants of which can affect the level of expression or transcript isoforms. Many authors have shown the importance of polymorphisms in the gene promoters, intronic and other non-coding regions (e.g., [45–48]).

Recently, other authors found a region on ECA6 (at a distance of about 4.4 Mb from that discovered by us) that was associated with the PM of spermatozoa after thawing in stallions [49]. It contained the best-associated SNP in an intron within the sodium voltage-gated channel alpha subunit 8 (*SCN8A*) gene. The same study also revealed a suggestive SNP in an intergenic region near the NOVA alternative splicing regulator 1 (*NOVA1*) gene on ECA1.

In conclusion, we emphasize that for the effective reproduction in horse breeding it is important to know functional genes and genomic variants affecting stallion fertility and semen quality during cryopreservation [10,16–22]. The suggestive SNPs we detected here by GWAS can be relevant to the candidate genes *NME8*, *OR2AP1*, *OR6C4* and, possibly, *PHACTR4* that can be associated with sperm motility in males.

The detected horse candidate genes may functionally trot out effects of homologous genes in humans and other animals. The SNP markers and candidate genes we identified here for cryotolerance in sperm as well as the respective genome regions can be helpful in studying the biological processes underlying the formation and functioning of the reproductive system of stallions. Polymorphism in the found candidate genes can be involved in sperm motility, suggesting their further detailed investigation and potential use in horse reproduction and breeding programs.

## Figures and Tables

**Table 1:** Motility parameters in stallion semen samples before and after freezing in the 23 horse breeds studied

Breeds	No. of males	PM (%)	FPM (%)	DPM (%)
Akhal-Teke	2	48.5±3.5	32.2±16.4	16.3±12.9
Appaloosa	1	43.0	26.0	17.0
Arabian	35	62.9±2.4	33.4±2.9	29.5±2.1
Bashkir	1	35.0	10.6	24.4
Budyonny	2	55.5±33.5	36.2±34.6	19.3±1.1
Don	2	31.0±1.0	13.5±1.5	17.5±2.5
French Trotter	7	77.1±5.0	44.3±6.7	32.8±3.1
German Warmblood	2	52.5±9.5	25.0±13.0	27.5±3.5
Hanoverian	1	59.2±9.6	32.3±8.0	26.9±3.4
Heavy Draft Crossbreds	3	58.5±6.5	16.5±3.5	42.0±3.0
Holsteiner	3	59±19.5	30.0±13.0	29±6.5
Karabakh	1	42.0	20.3	21.7
Orlov Trotter	4	62.2±3.6	33.8±4.2	28.4±2.4
Rhenish German Coldblood	1	49.0	38.3	10.7
Russian Heavy Draft	1	10.0	5.8	4.2
Russian Riding	1	40	10	30
Selle Français	1	61.0	46.0	15.0
Soviet Heavy Draft	11	58.1±4.0	30.0±5.2	28.1±2.5
Standardbred	6	63.7±4.4	36.1±4.1	27.6±3.7
Tersk	1	75.0	44.6	30.4
Thoroughbred	1	35.0	13.7	21.3
Trakehner	6	67.5±2.3	42.9±2.5	24.6±3.3
Welsh Pony	3	70.7±12.9	36.3±10.5	34.4±5.3

PM, progressive motility; FPM, post-thaw forward progressive motility; DPM, difference between PM and FPM values.

**Table 2:** Single nucleotide polymorphisms (SNPs) associated with difference between semen motility before and after thawing, their minor allele frequency (MAF), and respective candidate genes

SNPs	Chromosome	SNP position	p-value	Motif	MAF (allele)	Location	Candidate genes
rs1141327473	ECA4	8415448	1.96E-06	T/C	0.48 (C)	Intron	*NME8*
rs1149048772	ECA6	74273805	3.60E-08	T/C	0.12 (C)	Intergenic region	*OR2AP1, OR6C4*

*NME8*, NME/NM23 family member 8; *OR2AP1*, olfactory receptor family 2 subfamily AP member 1; *OR6C4*, olfactory receptor family 6 subfamily C member 4.

**Table 3:** Motility indices of native sperm and assessment of the effect of cryopreservation on semen of stallions of various SNP genotypes

Stallion SNP genotypes	No. of males	PM (%)	FPM (%)	DPM (%)
rs1149048772 – CC	3	26.66±11.66^d^	13.33±6.69	13.33±4.98^a^
rs1149048772 – CT	19	51.75±5.16	21.38±3.88	30.38±2.17^b^
rs1149048772 – TT	74	61.92±1.76^e^	21.80±1.01	40.11±1.08^c^
rs1141327473 – CC	18	43.71±4.21^f^	16.71±2.45^l^	27.00±2.42^i^
rs1141327473 – CT	45	60.08±2.64^g^	22.47±1.65^m^	37.61±1.56^j^
rs1141327473 – TT	33	65.42±2.39^h^	22.47±1.46^n^	42.95±1.44^k^

SNP, single nucleotide polymorphism; PM, progressive motility; FPM, post-thaw forward progressive motility; DPM, difference between PM and FPM values.

Significant differences:

a–k p<0.01.

**Table 4:** Frequency of minor alleles (MAF) in significant SNPs by breed

Breed	No. of males	MAF (C) in SNPs	

		rs1149048772	rs1141327473
Akhal-Teke	2	0	0.75
Appaloosa	1	0	0.5
Arabian	35	0.1	0.39
Bashkir	1	0.5	0.5
Budyonny	2	0.25	0.5
Don	2	0.5	0.5
French Trotter	7	0.07	0.28
German Warmblood	2	0.25	0.25
Hanoverian	1	0	0
Heavy Draft Crossbreds	3	0.33	1
Holsteiner	3	0.17	0.33
Karabakh	1	0.5	1
Orlov Trotter	4	0	0.5
Rhenish German Coldblood	1	0	0.5
Russian Heavy Draft	1	1	1
Russian Riding	1	0	0.5
Selle Français	1	1	0.5
Soviet Heavy Draft	11	0.05	0.45
Standardbred	6	0.08	0
Tersk	1	0	0
Thoroughbred	1	0	1
Trakehner	6	0.17	0.25
Welsh Pony	3	0	0.83

SNP, single nucleotide polymorphism.

## SUPPLEMENTARY MATERIAL

**Supplementary Figure S1.** Distribution of the studied 23 horse breeds as built in the web tools Phantasus ([32]; A) and ClustVis ([33]; B) using sperm motility data before and after freezing.
