# Supplementary material for: Genome-wide association study for frozen-thawed sperm motility in stallions across various horse breeds
Source: Anim Biosci. 2022 Mar 3;35(12):1827–38. doi: 10.5713/ab.21.0504 (PMC9659452; doi:10.5713/ab.21.0504)

Supplementary information

Genome-wide association study for frozen-thawed sperm motility in stallions across various horse breeds

Elena V. Nikitkina[1], Natalia V. Dementieva[1], Yuri S. Shcherbakov[1], Mikhail M. Atroshchenko[2], Andrei A. Kudinov[1], Oleg I. Samoylov[1], Marina V. Pozovnikova[1], Artem P. Dysin[1], Anna A. Krutikova[1], Artem A. Musidray[1], Olga V. Mitrofanova[1], Kirill V. Plemyashov[1], Darren K. Griffin[3], and Michael N. Romanov[3,4*]

Supplementary figure 1

Supplementary Figure

Supplementary Figure S1. Distribution of the studied 23 horse breeds as built in the web tools Phantasus ([32]; A) and ClustVis ([33]; B) using sperm motility data before and after freezing. (A) PCA plot. Unit variance scaling is applied to rows; SVD with imputation is used to calculate principal components (PC). *X* and *Y* axes show PC1 and PC2 that explain 71.8% and 28.2% of the total variance, respectively. N = 23 data points (breeds). (B) Heatmap and clustering trees, with rows being centered and unit variance scaling being applied to rows. Both three rows and 23 columns are clustered using Euclidean distance and average linkage, with tightest cluster first for tree ordering.

(A)

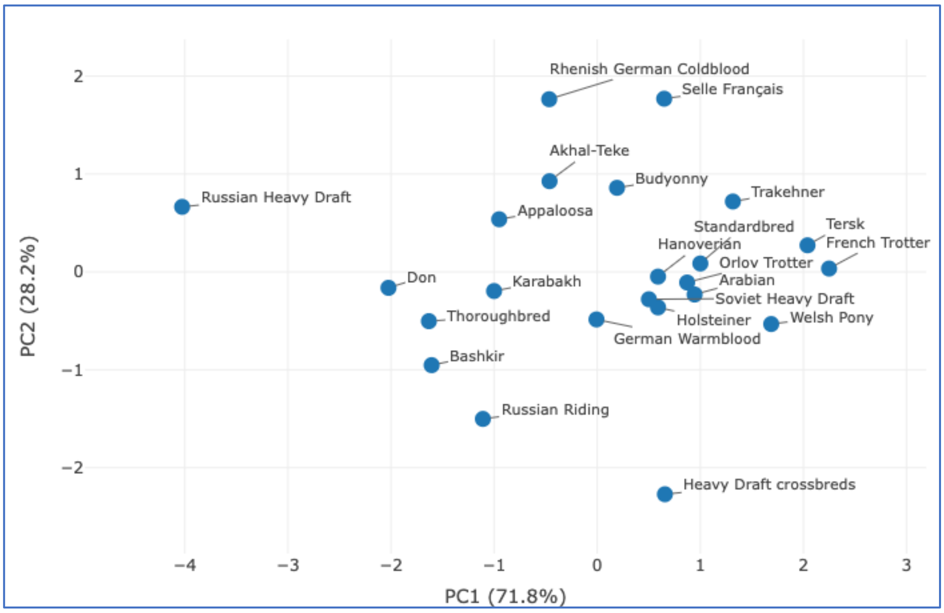

(B)

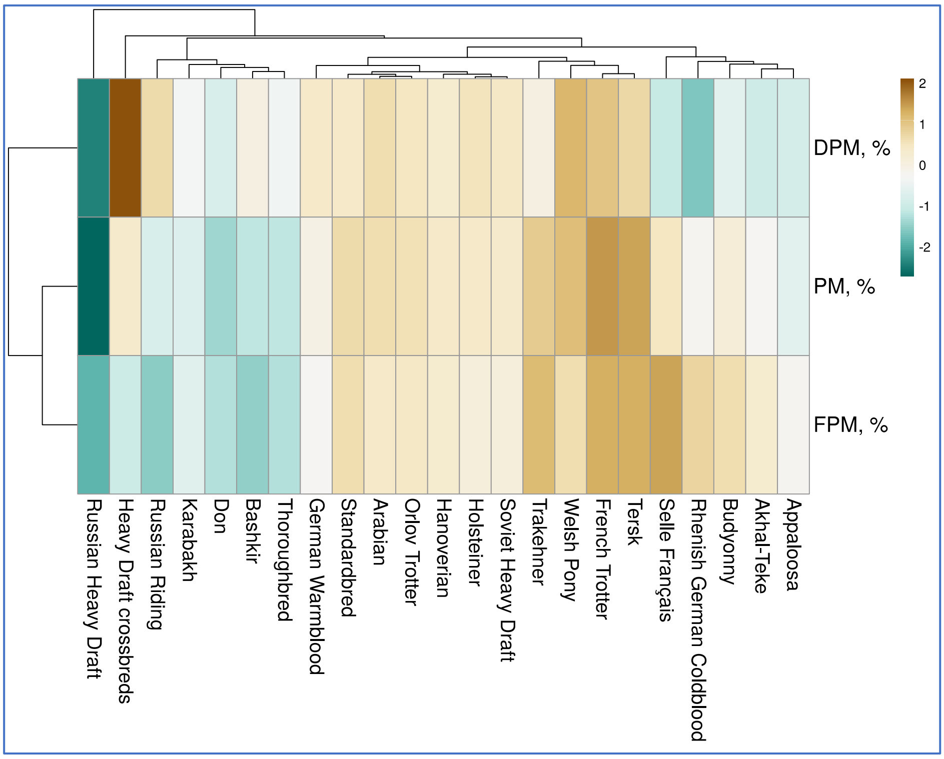

Supplement: Supplementary file 1 [file ab-21-0504-suppl.pdf]
